# Anti-rods/rings autoantibody seropositivity does not affect response to telaprevir treatment for chronic hepatitis C infection

**DOI:** 10.1007/s13317-016-0087-9

**Published:** 2016-11-14

**Authors:** S. John Calise, Nicola Bizzaro, Thuy Nguyen, Danila Bassetti, Brunetta Porcelli, Paolo Almi, Giuseppina Barberio, Giampaola Pesce, Minoru Satoh, Edward K. L. Chan

**Affiliations:** 1 Department of Oral Biology, University of Florida, 1395 Center Drive, Gainesville, FL 32610-0424 USA; 2 Laboratorio di Patologia Clinica, Ospedale San Antonio, Tolmezzo, Italy; 3 Microbiologia e Virologia, Ospedale Santa Chiara, Trento, Italy; 4 Dipartimento Biotecnologie Mediche, UOC Laboratorio Patologia Clinica, Azienda Ospedaliera Universitaria Senese, Università degli Studi di Siena, Siena, Italy; 5 UOC Malattie Infettive ed Epatologia, Azienda Ospedaliera Universitaria Senese, Siena, Italy; 6 Medicina di Laboratorio, Ospedale Regionale, Treviso, Italy; 7 Dipartimento di Medicina Interna e Specialità Mediche, Università degli Studi di Genova, Genova, Italy; 8 Department of Clinical Nursing, University of Occupational and Environmental Health, Kitakyushu, Japan

**Keywords:** Direct-acting antivirals, Inosine monophosphate dehydrogenase, Interferon-α, Ribavirin, Rods and rings, Telaprevir

## Abstract

**Purpose:**

Autoantibodies to intracellular ‘rods and rings’ structures (anti-rods/rings or anti-RR) are strongly associated with hepatitis C (HCV) patients treated with interferon-α/ribavirin (IFN/RBV) and are linked with non-responsiveness to IFN/RBV or relapse, especially in Italian patients. This is the first study to determine whether there is any correlation of anti-RR with non-responsiveness to IFN/RBV treatment in patients also treated with telaprevir (TPV), one of several new therapies for chronic HCV recently implemented.

**Methods:**

From 2013 to 2014, 52 HCV-infected patients were treated with IFN/RBV and TPV at five Italian clinics. Patient sera were collected and analyzed by indirect immunofluorescence for the presence of anti-RR antibodies. Patients were classified as anti-RR positive or anti-RR negative, and then various biological and clinical variables were analyzed to compare the two groups, including gender, age, HCV genotype, previous IFN/RBV treatment, and IFN/RBV/TPV treatment outcome.

**Results:**

Of these 52 HCV patients treated with IFN/RBV/TPV, 10/32 (31%) who previously received IFN/RBV were anti-RR positive, compared to 0 of 20 treatment-naïve patients. Anti-RR-positive patients relapsed more than anti-RR-negative patients (3/10, 30% vs. 2/42, 5%; *p* < 0.05). However, zero anti-RR-positive patients were non-responsive, and frequencies of sustained virological response were similar (anti-RR positive: 7/10, 70% vs. anti-RR negative: 33/42, 79%).

**Conclusions:**

Overall, the data suggest that anti-RR seropositivity is not associated with resistance to TPV treatment in this patient cohort, but monitoring anti-RR-positive patients for relapse within the first 6 months after treatment may be useful.

## Introduction

Chronic hepatitis C (HCV) infection is associated with the production of autoantibodies, including organ-specific autoantibodies directed against targets in the thyroid, adrenal cortex, pancreatic islet cells, and gastric parietal cells, and non-organ-specific autoantibodies such as antinuclear, anti-smooth muscle, anti-mitochondrial, anti-liver/kidney microsomal, and anti-neutrophil cytoplasmic antibodies [1–8]. Recent studies have also demonstrated a link between HCV and the production of autoantibodies targeting intracellular filamentous structures termed ‘rods and rings’ (RRs) [9–16]. In most studies, anti-rods/rings (anti-RR) seropositivity appears to be almost exclusive to HCV patients treated with interferon-α and ribavirin combination therapy (IFN/RBV) and is rarely seen in treatment-naïve HCV patients or other disease groups. However, anti-RR has been observed in one hepatitis B patient [11], one systemic lupus erythematosus patient [17], and healthy individuals with no previous IFN/RBV treatment [17]. In cultured cells, RRs are composed of inosine 5′-monophosphate dehydrogenase (IMPDH), and/or cytidine 5′-triphosphate synthase under certain conditions [9, 18, 19]. RRs tend to assemble when de novo purine biosynthesis is inhibited and guanine nucleotide levels become depleted [20–25]. Many patients with anti-RR react with IMPDH, which is inhibited by direct binding to RBV and appears to be the major autoantigen in RRs [9, 26, 27]. Although no mechanistic evidence suggesting that anti-RR autoantibody contributes to resistance to IFN/RBV therapy has yet been reported, previous studies showed that anti-RR antibodies were more prevalent in patients who did not respond to therapy or relapsed, when compared to sustained responders [10, 15]. Additionally, non-responsive or relapsing patients had higher anti-RR titers, suggesting that anti-RR positivity may be indicative of poor treatment outcomes [28]. In recent years, direct-acting antivirals (DAAs), such as telaprevir (TPV), have been developed for chronic HCV infection in an effort to reduce therapy duration and increase drug tolerability, while also improving patient outcomes. Currently, TPV is included with IFN/RBV as a triple therapy. Here, we examine the relationship between anti-RR and treatment outcomes in a cohort of Italian patients treated with IFN/RBV and telaprevir.

## Methods

### Patient and treatment information

From 2013 to 2014, 52 HCV-infected patients were treated with IFN/RBV and TPV at five Italian clinics located at the (1) Ospedale San Antonio, Tolmezzo, (2) Ospedale Santa Chiara, Trento, (3) Università degli Studi di Siena, (4) Ospedale Regionale, Treviso, and (5) Università degli Studi di Genova. Dosages depended on patient weight (75 kg discriminating weight) and were typically administered as follows: 80–180 µg weekly pegylated interferon-α, 600–1400 mg daily ribavirin, and 2250 mg daily TPV. Patients were classified as: not responsive to therapy (HCV RNA still detectable at week 24 of therapy), relapsed (HCV RNA detectable after the end of treatment in patients with previous virological response), or responsive to therapy (HCV RNA not detectable in the 24 weeks after the completion of therapy). The study conforms to the Institutional Review Board requirements in all institutions.

*Informed consent* Informed consent was obtained from all individual participants included in the study. All patients provided written informed consent to receive IFN/RBV/TPV and permission for use of their medical records for this study.

### Antinuclear antibody indirect immunofluorescence assay (ANA-IIF)

Anti-rods/rings in patient sera were detected by indirect immunofluorescence, using NOVA Lite HEp-2 ANA substrate (INOVA Diagnostics, San Diego, CA: 508100) as previously described [10]. Staining patterns of test sera were compared to staining of human prototype anti-RR serum It2006 used in previous studies [9, 10]. It2006 and all anti-RR-positive sera described in this study correctly recognize the rods and rings ANA pattern, designated as pattern AC-23 by the International Consensus on ANA Patterns (ICAP) [29]. All sera were tested at a dilution of 1:80 in PBS as previously described [30]. For anti-RR-positive patients who also had serial samples available, anti-RR end point titers were determined using twofold serial dilution of sera in PBS, with a starting dilution of 1:80 and ending dilution of 1:1280. Anti-RR positivity and titers were independently validated by two trained individuals (S.J.C. and T.N.). End point titer was defined by more than 50% of cells containing detectable RR staining. Donkey anti-human IgG conjugated to DyLight 488 (Jackson ImmunoResearch, West Grove, PA) diluted 1:100 in PBS was used to detect autoantibody staining. Fluorescent images were captured with a Zeiss Axiovert 200 M microscope fitted with a Zeiss AxioCam MRm camera using a 40× (0.75 NA) objective (Carl Zeiss Microscopy, Jena, Germany).

### Statistical analysis

Biological and clinical variables analyzed for statistical significance include: gender, age, HCV genotype, previous treatment with IFN/RBV (prior to beginning of TPV regimen), and treatment outcome (see Table 1). Mann–Whitney *U* test was used to compare different groups containing continuous data, and Fisher’s exact test or the Fisher–Freeman–Halton exact test was used for categorical data. Differences were considered statistically significant if *p* < 0.05. Mann–Whitney *U* test and Fisher’s exact test were performed using GraphPad Prism 5.0 (GraphPad Software, La Jolla, CA). Fisher–Freeman–Halton test was performed using StatXact 10 (Cytel, Cambridge, MA).

**Table 1:** Summary of anti-RR autoantibody reactivity in HCV patients treated with interferon-α/ribavirin and telaprevir

Parameters	Total patients (*n* = 52)	Anti-RR-positive patients (*n* = 10)	Anti-RR-negative patients (*n* = 42)	*p* value
Male	36 (69%)	7 (70%)	29 (69%)	NS
Female	16 (31%)	3 (30%)	13 (31%)	NS
Age (years) ± SD	54 ± 9	53 ± 14	54 ± 8	NS
Genotype 1a^#^	12 (23%)	3 (30%)	9 (21%)	NS
Genotype 1b^#^	39 (75%)	7 (70%)	32 (76%)	NS
Previous IFN/RBV	32 (62%)	10 (100%)	22 (52%)	<0.01
Treatment outcome				
SVR, no side effects	35 (67%)	6 (60%)	29 (69%)	NS
SVR, but side effects	5 (10%)	1 (10%)	4 (10%)	NS
SVR, combined	40 (77%)	7 (70%)	33 (79%)	NS
Relapse	5 (10%)	3 (30%)	2 (5%)	<0.05
No response	7 (13%)	0 (0%)	7 (17%)	NS
Fisher–Freeman–Halton exact test				
Anti-RR positive vs. anti-RR negative, with SVR separated				NS
Anti-RR positive vs. anti-RR negative, with SVR combined				<0.05

Values presented as *n* (%) unless otherwise indicated

*Anti-RR* anti-rods/rings autoantibody, *IFN/RBV* interferon-α/ribavirin therapy, *NS* not statistically significant (*p* > 0.05), *SD* standard deviation, *SVR* sustained virological response

^#^One patient without anti-RR was genotype 3b

## Results and discussion

The goal of this study was to probe for an association between the presence of anti-RR autoantibody and treatment outcome in a cohort of 52 Italian HCV patients treated with the new DAA telaprevir. This is the first study to examine anti-RR in patients treated with any of the recently approved DAAs. It must be pointed out that, in general, the availability of patients for studies of anti-RR antibody is naturally limited by the low prevalence of this autoantibody response. Although it has been reported that 20–40% of HCV patients treated with IFN/RBV produce anti-RR autoantibodies, this response is very rarely observed in treatment-naïve HCV patients or other disease groups [10, 11, 14–16, 31].

**Fig. 1:**
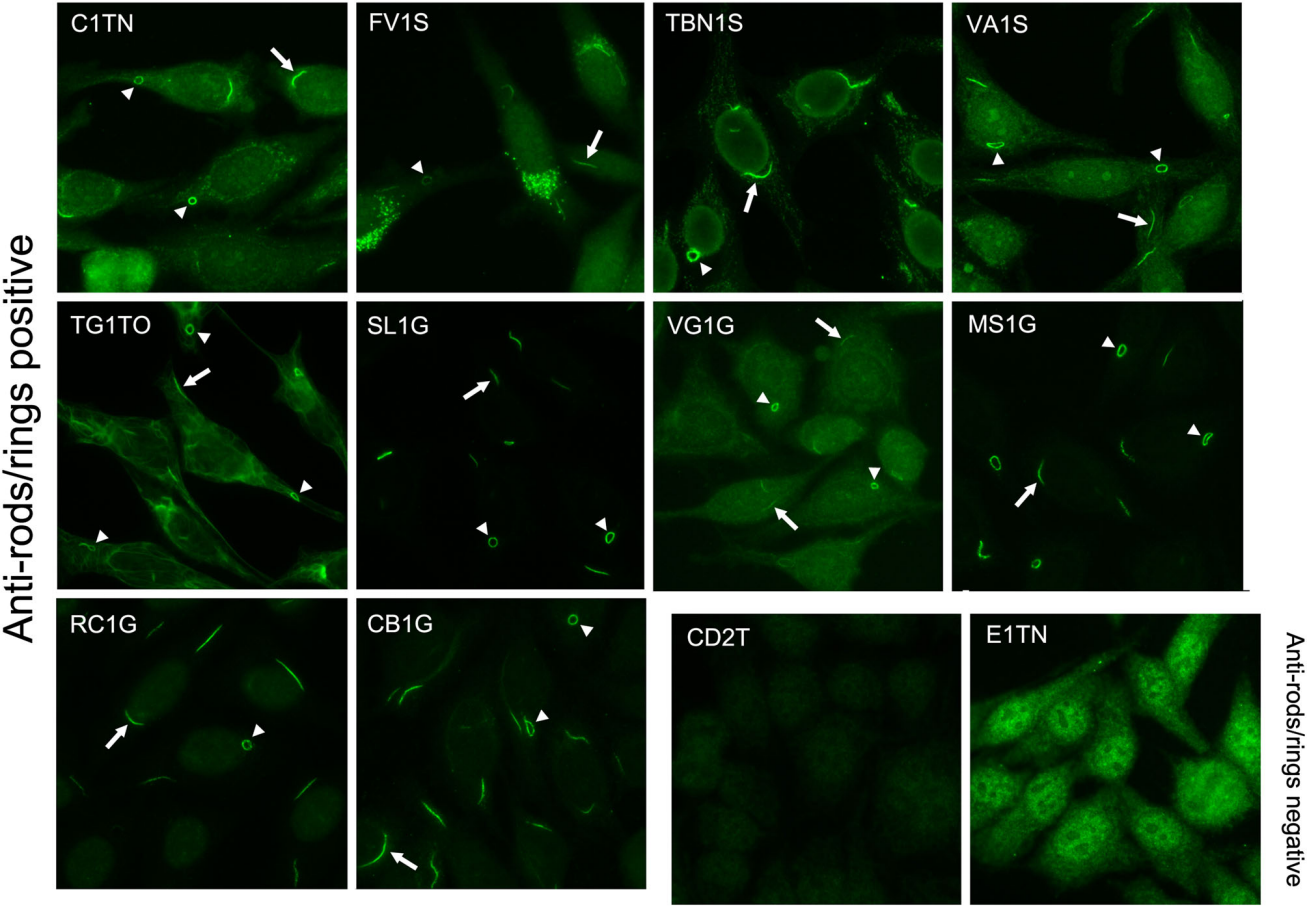
Anti-rods/rings seropositivity detected by the ANA-IIF assay. Fifty-two Italian HCV patients were subjected to the antinuclear antibody indirect immunofluorescence (ANA-IIF) assay. Anti-rods/rings autoantibody was detected in 10 of 52 patients (19%), shown by the ‘rods and rings’ ANA pattern in patient codes C1TN, FV1S, TBN1S, VA1S, TG1TO, SL1G, VG1G, MS1G, RC1G, and CB1G. *Arrows* point to examples of rods, which are most often observed in cytoplasmic and perinuclear regions (see C1TN, RC1G, or CB1G for examples of perinuclear rods). *Arrowheads* point to rings, which may sometimes appear twisted into a “hairpin” shape, as in the *bottom left corner* of the *panel* TG1TO. CD2T and E1TN are negative for anti-rods/rings and are included for comparison. CD2T contains no detectable autoantibody reactivity, while E1TN displays the nuclear speckled ANA pattern. All *panels* shown are of sera tested at 1:80 dilution and detected by donkey anti-human IgG conjugated to DyLight 488. All images were taken with a 40× objective

A total of 52 HCV-infected patients were treated with IFN/RBV and TPV. Thirty-six patients (69%) were male and 16 (31%) were female, with a mean age of 54 ± 9 years. Twelve patients (23%) had genotype 1a, 39 patients (75%) had genotype 1b, and one patient (2%) had genotype 3b. Thirty-two patients (62%) had been previously treated with IFN/RBV prior to being put on TPV. Patient demographics are included in Table 1. All 52 patients were assayed for the presence of anti-RR according to standard antinuclear antibody indirect immunofluorescence (ANA-IIF) protocols using HEp-2 cells as a substrate. ANA-IIF analysis revealed that 10 out of 52 patients (19%) were positive for anti-RR at a dilution of 1:80. Figure 1 displays the images of the ‘rods and rings’ staining pattern from all ten anti-RR-positive patients (patient codes C1TN, FV1S, TBN1S, VA1S, TG1TO, SL1G, VG1G, MS1G, RC1G, and CB1G). Anti-RR-negative patients CD2T and E1TN are also shown for comparison. Twenty-one out of 52 patients had serial collections of sera available (104 total sera for 52 patients), representing multiple visits to the clinic over time periods ranging from 2 weeks to 13 months. Anti-RR status did not change over time in any of these 21 patients (i.e., anti-RR-positive patients remained positive and negative patients remained negative) and anti-RR titers did not significantly change in positive patients. Accordingly, for purposes of statistical analysis, patients were simply considered either positive or negative, and serial collections were not taken into consideration.

Patients were divided into two main groups, anti-RR-positive (*n* = 10) and anti-RR-negative (*n* = 42), for subsequent statistical analysis. These groups were compared based on several demographic, clinical, and serological parameters to determine any differences between anti-RR-positive and anti-RR-negative patients (Table 1). There was no significant difference observed between the two groups when comparing gender, age, or HCV genotype. Both patient groups were also categorized into four different treatment outcome groups: (1) sustained virological response (SVR) with no side effects, (2) initial SVR but therapy was discontinued due to side effects, (3) relapse within 6 months of treatment, and (4) no response to treatment. SVR patients with or without side effects were all determined to be SVR at the same time point (after 1 month, according to international guidelines). When the Fisher–Freeman–Halton exact test was performed to compare anti-RR-positive vs. anti-RR-negative patient groups based on all four treatment outcome parameters, no significant difference was observed. However, additional Fisher–Freeman–Halton analysis with all SVR patients combined (regardless of side effects) resulted in a statistically significant *p* value <0.05 (Table 1, bottom row). Thus, our data indicate that anti-RR-positive and anti-RR-negative patients in this cohort appear to differ with regard to distribution of treatment outcomes. The most notable difference is that anti-RR-positive patients were more likely to relapse than anti-RR-negative patients (*p* < 0.05). Despite the increase in relapse, 0 of the 10 anti-RR-positive patients were non-responsive to therapy, compared to 7 of the 42 (17%) anti-RR-negative patients (*p* = 0.32). Additionally, there was no significant difference in SVR rates between both groups. SVR rates were compared with patients who experienced side effects and those who did not separated as different outcomes (“SVR, but side effects” and “SVR, no side effects”) or with all SVR patients combined (“SVR, combined”), but both analyses showed no significant difference between anti-RR-positive and anti-RR-negative patients.

We also discovered that anti-RR can be detected years after treatment with IFN/RBV. Two of the ten anti-RR-positive patients had serum collected both prior to and after IFN/RBV/TPV therapy was initiated. All samples from both patients were positive for anti-RR, initially suggesting that these patients may have been positive with no previous IFN/RBV treatment. However, after careful examination of medical records, it was determined that both patients had received IFN/RBV more than a decade prior to treatment with TPV. Patient TBN1S was diagnosed with liver cirrhosis in 2000 and treated with IFN/RBV from 2000 to 2002, but treatment was eventually discontinued due to side effects. In 2014, TBN1S began receiving IFN/RBV again with addition of TPV, but relapsed within 6 months. Patient VA1S previously received IFN/RBV for 6 months in 2003. Eleven years later, in 2014, the patient began receiving IFN/RBV again with addition of TPV, but therapy was discontinued due to side effects. Despite the lack of exposure to IFN/RBV for more than 10 years, both patients remained positive for anti-RR autoantibody, and VA1S even remained positive down to 1:1280 dilution. We speculate that long-lived plasma cells might be responsible for the long-term presence of anti-RR antibody in these patients. Previous studies have suggested that anti-RR titer increases throughout the duration of therapy, but declines upon cessation of treatment [11, 12, 15, 31]. To our knowledge, this is the first report of long-lived anti-RR autoantibody.

Previous studies established a strong association between anti-RR and IFN/RBV therapy [10–12, 14, 15], such that we previously described anti-RR as a drug-induced autoantibody [13]. Additionally, prolonged exposure to IFN/RBV increases the likelihood of anti-RR autoantibody production [11, 12, 15]. Our data support these findings, considering that 10 out of 32 (31%) patients previously treated with IFN/RBV were anti-RR positive, compared to 0 out of 20 patients who had not previously received IFN/RBV. In terms of treatment outcome, previous reports have indicated a link between anti-RR and non-responsiveness or relapse in American and Italian HCV patient cohorts [10, 15, 28]. In the current study with a new cohort of Italian patients, we again found that anti-RR seropositivity was associated with increased frequency of relapse. Interestingly, the frequency of non-responsiveness appeared to be decreased in anti-RR-positive patients (0/10, 0%) compared to anti-RR-negative patients (7/42, 17%), despite the opposite trend in relapse. When patients with no previous IFN/RBV treatment are removed from analysis, the trend remains similar, with non-responsiveness occurring in 0/10 (0%) of anti-RR-positive patients compared to 6/16 (27%) of anti-RR-negative patients (*p* = 0.14). Overall, 19% (10/52) of HCV patients in this study were positive for anti-RR, which is similar to the rates observed in previous studies [10, 11, 14–16]. Importantly, the addition of TPV to the IFN/RBV regimen did not induce a new anti-RR response in any patients; anti-RR-negative patients previously treated with IFN/RBV did not become positive after TPV was included in the regimen. Likewise, anti-RR titers did not significantly change after addition of TPV in anti-RR-positive patients with serial samples available. In all, our data suggest that inclusion of TPV in the IFN/RBV regimen for the treatment of chronic HCV does not alter the production of anti-RR autoantibody. However, based on the statistically significant increase in relapse rate, it may be useful to carefully monitor anti-RR-positive patients during and for 6 months after IFN/RBV/TPV therapy. While our study is limited by the number of available patients, the data indicate that anti-RR seropositivity does not affect the response to TPV treatment for chronic HCV.

## Abbreviations

ANA: Antinuclear antibody
anti-RR: Anti-rods/rings autoantibody
DAAs: Direct-acting antivirals
HCV: Hepatitis C
IFN: Interferon-α
IFN/RBV: Interferon-α and ribavirin therapy
IMPDH: Inosine 5′-monophosphate dehydrogenase
RBV: Ribavirin
RRs: Rods and rings
SVR: Sustained virological response
TPV: Telaprevir
